# *trans*-Bis(acetonitrile-κ*N*)bis­{1,2-bis­[bis­(3-hydroxy­prop­yl)phosphino]ethane-κ^2^ *P*,*P*′}iron(II) dichloride

**DOI:** 10.1107/S1600536809021758

**Published:** 2009-06-17

**Authors:** J. W. Gohdes, Lev N. Zakharov, David R. Tyler

**Affiliations:** a Department of Chemistry, Pacific University, 2043 College Way, Forest Grove, OR, USA; b Department of Chemistry, 1253 University of Oregon, Eugene, Oregon 97403-1253, USA

## Abstract

In the title compound, [Fe(CH_3_CN)_2_(C_14_H_32_O_4_P_2_)_2_]Cl_2_, the Fe^II^ atom lies on a crystallographic inversion center and has a distorted *trans*-FeN_2_P_4_ octa­hedral coordination environment arising from two *P*,*P*′-bidentate 1,2-bis­[bis­(3-hydroxy­prop­yl)phosphino]ethane ligands in the equatorial plane and two acetonitrile mol­ecules in the axial positions. One of the pendant –(CH_2_)_3_OH groups of the ligand is disordered over two sets of sites in a 0.597 (5):0.403 (5) ratio. In the crystal, O—H⋯Cl and O—H⋯O hydrogen bonding helps to establish the packing.

## Related literature

For related compounds containing bidentate phosphine ligands, see: Gilbertson *et al.* (2007 ▶); Miller *et al.* (2002 ▶); Martins *et al.* (1998 ▶); Barron *et al.* (1987 ▶); George *et al.* (1997 ▶); Edwards *et al.* (2006 ▶). For reference structural data, see: Allen *et al.* (1987 ▶).
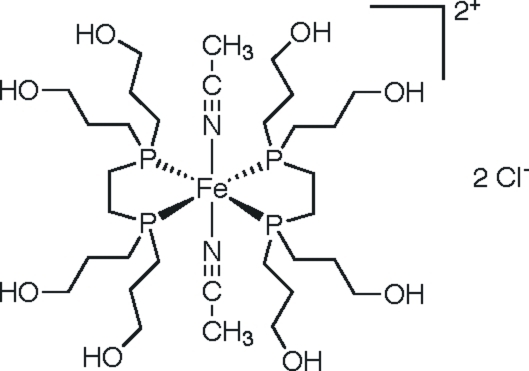


## Experimental

### 

#### Crystal data

[Fe(C_2_H_3_N)_2_(C_14_H_32_O_4_P_2_)_2_]Cl_2_
*M* *_r_* = 861.53
Orthorhombic, 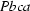

*a* = 18.3024 (12) Å
*b* = 11.5220 (8) Å
*c* = 19.8413 (13) Å
*V* = 4184.1 (5) Å^3^
*Z* = 4
Mo *K*α radiation
μ = 0.69 mm^−1^
*T* = 173 K
0.22 × 0.18 × 0.17 mm

#### Data collection

Bruker APEX CCD diffractometer
Absorption correction: multi-scan (*SADABS*; Bruker, 2000 ▶) *T*_min_ = 0.863, *T*_max_ = 0.892
24288 measured reflections
4571 independent reflections
4122 reflections with *I* > 2σ(*I*)
*R*_int_ = 0.024

#### Refinement

*R*[*F* ^2^ > 2σ(*F* ^2^)] = 0.035
*wR*(*F* ^2^) = 0.092
*S* = 1.04
4571 reflections
269 parameters
7 restraints
H atoms treated by a mixture of independent and constrained refinement
Δρ_max_ = 0.88 e Å^−3^
Δρ_min_ = −1.01 e Å^−3^

### 

Data collection: *SMART* (Bruker, 2000 ▶); cell refinement: *SAINT* (Bruker, 2000 ▶); data reduction: *SAINT*; program(s) used to solve structure: *SHELXTL* (Sheldrick, 2008 ▶); program(s) used to refine structure: *SHELXTL*; molecular graphics: *SHELXTL*; software used to prepare material for publication: *SHELXTL*.

## Supplementary Material

Crystal structure: contains datablocks I, global. DOI: 10.1107/S1600536809021758/hb2994sup1.cif

Structure factors: contains datablocks I. DOI: 10.1107/S1600536809021758/hb2994Isup2.hkl

Additional supplementary materials:  crystallographic information; 3D view; checkCIF report

## Figures and Tables

**Table 1:** Selected bond lengths (Å)

Fe1—N1	1.9077 (14)
Fe1—P1	2.2884 (4)
Fe1—P2	2.3049 (4)

**Table 2:** Hydrogen-bond geometry (Å, °)

*D*—H⋯*A*	*D*—H	H⋯*A*	*D*⋯*A*	*D*—H⋯*A*
O1—H1*O*⋯Cl1	0.928 (17)	2.126 (18)	3.0493 (16)	173 (3)
O2—H2*O*⋯Cl1^i^	0.976 (18)	2.23 (2)	3.1777 (19)	164 (3)
O3—H3*O*⋯O1^i^	0.924 (17)	1.841 (18)	2.741 (2)	164 (2)
O4—H4*O*⋯Cl1^ii^	0.98 (2)	1.95 (2)	2.931 (6)	177 (3)
O4*A*—H4*OA*⋯Cl1^ii^	0.98 (2)	2.84 (11)	3.490 (10)	125 (9)

Symmetry codes: (i) 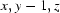; (ii) 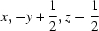.

**Fig. 1.**
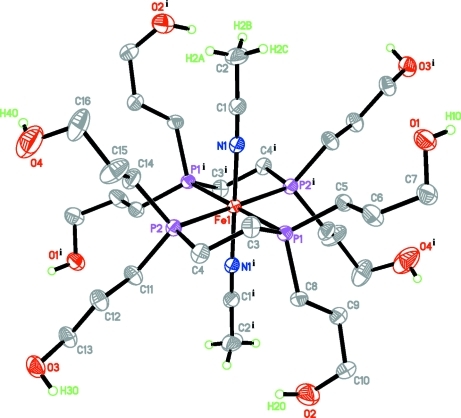
The stucture of the [Fe(DHPrPE)2(CH3CN)2]2+ cation in (I) with 50% probability displacement elipsoids. Only the H atoms in the –OH and –CH3 groups and only one position of the disordered hydroxypropyl group are shown for clarity. Symmetry code (i): –x, –y, –z.

## supplementary crystallographic information

### Comment

The bidentate phosphine 1,2-bis[di(3-hydroxypropyl)phosphino]ethane (DHPrPE) was developed as a water soluble ligand for use in making iron complexes capable of binding dinitrogen and hydrogen (Miller *et al.*, 2002). It was found that the hydroxypropyl groups were non-innocent in reactions with iron(II), and a stable complex was isolated in which the chelating phosphine ligands are tridentate and coordinate through one of the hydroxypropyl groups in addition to both phosphines. This results in a coordinatively saturated complex where the alcohols ligands are *cis* to one another. The current work shows that addition of acetonitrile to this species results in the rearrangement to the *trans* geometry.

The stucture of the cation [Fe(DHPrPE)_2_(CH_3_CN)_2_]^2+^ in the title compound, (I), is shown in Fig. 1. The four phosphine donors from the DHPrPE ligands form a square planar arrangement around the iron atom and the two coordinated acetonitrile ligands occupy the *trans* axial sites to form a distorted octahedral geometry around the iron. Such *trans* bis acetonitrile complexes of iron(II) with bidentate phosphines are not uncommon. The first reported structure was of the DMPE analog (Barron *et al.* 1987). An examination of similar compounds (George *et al.* 1997, Martins *et al.* 1998, Gilbertson *et al.* 2007 and Edwards *et al.* 2006) shows there are minor variations within the primary coordination sphere of all of these complexes. The Fe—N bond distances vary from 1.895 to 1.917 Å; the Fe—P bond distances vary from 2.255 to 2.3032 Å and the P—Fe—P bite angles are between 84.0° and 85.5°. The Fe—P distances in the title compound, 2.2883 (5) and 2.3044 (5) Å, are at the high end of the expected range while the bite angle of 84.2° is at the low end of the range, indicating significant steric crowding around the iron center.

### Experimental

The title compound was prepared by dissolving 60 mg of iron(II)chloride tetrahydrate (0.30 mmole) and 200 mg of 1,2-bis[di(3-hydroxypropyl)phosphino]ethane (0.61 mmole) in 3.0 ml of methanol to give a dark purple solution. After addition of 2.0 ml of acetonitrile, the solution slowly turned orange indicating formation of the title complex. Addition of diethylether and filtration yielded 215 mg (90%) of the title compound as an orange, crystalline powder and gave a single resonance in the ^31^P{^1^H} NMR spectrum at 61.4 p.p.m..  Yellow blocks of (I) were grown by vapor diffusion of diethylether into a 3:1 methanol/acetonitrile solution of the complex.

### Refinement

One of the hydroxypropyl side chains is disordered over two postions in ratio 60/40. The disordered fragment was refined with the same displacement parameters for atoms in each disordered positions. The H atoms on the acetonitrile methyl groups and the alcohol groups except for the disordered one were located on residual density map and refined with isotropic thermal parameters and with restrictions; the average O—H distance of 0.967 Å (Allen *et al.* 1987) was used as a target for corresponding O—H bonds. All H atoms in –CH_2_ groups were positioned geometrically and refined as riding with C—H = 0.99 Å and *U*_iso_(H)=1.2 *U*_eq_(C).

### Crystal data

**Table:**

[Fe(C_2_H_3_N)_2_(C_14_H_32_O_4_P_2_)_2_]Cl_2_	*F*(000) = 1840
*M**_r_* = 861.53	*D*_x_ = 1.368 Mg m^−^^3^
Orthorhombic, *P**b**c**a*	Mo *K*α radiation, λ = 0.71073 Å
Hall symbol: -P 2ac 2ab	Cell parameters from 5018 reflections
*a* = 18.3024 (12) Å	θ = 2.3–27.0°
*b* = 11.5220 (8) Å	µ = 0.69 mm^−^^1^
*c* = 19.8413 (13) Å	*T* = 173 K
*V* = 4184.1 (5) Å^3^	Block, yellow
*Z* = 4	0.22 × 0.18 × 0.17 mm

### Data collection

**Table:**

Bruker APEX CCD diffractometer	4571 independent reflections
Radiation source: fine-focus sealed tube	4122 reflections with *I* > 2σ(*I*)
graphite	*R*_int_ = 0.024
φ and ω scans	θ_max_ = 27.0°, θ_min_ = 2.1°
Absorption correction: multi-scan (*SADABS*; Bruker, 2000)	*h* = −22→23
*T*_min_ = 0.863, *T*_max_ = 0.892	*k* = −14→13
24288 measured reflections	*l* = −25→14

### Refinement

**Table:**

Refinement on *F*^2^	Primary atom site location: structure-invariant direct methods
Least-squares matrix: full	Secondary atom site location: difference Fourier map
*R*[*F*^2^ > 2σ(*F*^2^)] = 0.035	Hydrogen site location: inferred from neighbouring sites
*wR*(*F*^2^) = 0.092	H atoms treated by a mixture of independent and constrained refinement
*S* = 1.04	*w* = 1/[σ^2^(*F*_o_^2^) + (0.0451*P*)^2^ + 3.5311*P*] where *P* = (*F*_o_^2^ + 2*F*_c_^2^)/3
4571 reflections	(Δ/σ)_max_ = 0.001
269 parameters	Δρ_max_ = 0.88 e Å^−^^3^
7 restraints	Δρ_min_ = −1.01 e Å^−^^3^

### Special details

**Table:**

Geometry. All e.s.d.'s (except the e.s.d. in the dihedral angle between two l.s. planes)are estimated using the full covariance matrix. The cell e.s.d.'s are takeninto account individually in the estimation of e.s.d.'s in distances, anglesand torsion angles; correlations between e.s.d.'s in cell parameters are onlyused when they are defined by crystal symmetry. An approximate (isotropic)treatment of cell e.s.d.'s is used for estimating e.s.d.'s involving l.s. planes.
Refinement. Refinement of *F*^2^ against ALL reflections. The weighted *R*-factor *wR* and goodness of fit *S* are based on *F*^2^, conventional *R*-factors *R* are based on *F*, with *F* set to zero for negative *F*^2^. The threshold expression of *F*^2^ > σ(*F*^2^) is used only for calculating *R*-factors(gt) *etc*. and is not relevant to the choice of reflections for refinement. *R*-factors based on *F*^2^ are statistically about twice as large as those based on *F*, and *R*- factors based on ALL data will be even larger.

### Fractional atomic coordinates and isotropic or equivalent isotropic displacement parameters (Å^2^)

**Table:**

	*x*	*y*	*z*	*U*_iso_*/*U*_eq_	Occ. (<1)
Fe1	0.0000	0.0000	0.0000	0.01629 (10)	
Cl1	0.09382 (5)	0.65273 (5)	0.16061 (4)	0.0673 (2)	
P1	0.08435 (2)	0.04855 (4)	0.08033 (2)	0.01886 (11)	
P2	0.08545 (2)	−0.13647 (4)	−0.03165 (2)	0.01984 (11)	
O1	0.17032 (9)	0.42889 (12)	0.11886 (8)	0.0372 (3)	
O2	0.01646 (10)	−0.18587 (14)	0.26912 (9)	0.0484 (4)	
O3	0.15466 (8)	−0.58403 (12)	−0.01831 (8)	0.0334 (3)	
N1	0.04458 (8)	0.11038 (12)	−0.05870 (7)	0.0203 (3)	
C1	0.07048 (10)	0.17507 (16)	−0.09498 (9)	0.0252 (4)	
C2	0.10388 (16)	0.2552 (2)	−0.14267 (12)	0.0427 (6)	
C3	0.17298 (10)	0.00506 (16)	0.04511 (10)	0.0253 (4)	
H3A	0.1855	0.0542	0.0059	0.030*	
H3B	0.2119	0.0134	0.0794	0.030*	
C4	0.16574 (9)	−0.12177 (16)	0.02359 (9)	0.0245 (4)	
H4A	0.1599	−0.1718	0.0638	0.029*	
H4B	0.2103	−0.1465	−0.0008	0.029*	
C5	0.09772 (10)	0.19994 (15)	0.10533 (9)	0.0229 (4)	
H5A	0.0970	0.2487	0.0643	0.028*	
H5B	0.0559	0.2239	0.1337	0.028*	
C6	0.16840 (11)	0.22501 (16)	0.14399 (11)	0.0344 (5)	
H6A	0.1734	0.1686	0.1813	0.041*	
H6B	0.2106	0.2145	0.1134	0.041*	
C7	0.16982 (12)	0.34726 (17)	0.17253 (11)	0.0351 (5)	
H7A	0.1263	0.3600	0.2012	0.042*	
H7B	0.2139	0.3577	0.2008	0.042*	
C8	0.08669 (10)	−0.03375 (16)	0.15970 (9)	0.0249 (4)	
H8A	0.0756	−0.1158	0.1490	0.030*	
H8B	0.1374	−0.0313	0.1771	0.030*	
C9	0.03621 (13)	0.00366 (17)	0.21634 (10)	0.0342 (5)	
H9A	−0.0150	0.0000	0.2004	0.041*	
H9B	0.0470	0.0852	0.2285	0.041*	
C10	0.04449 (13)	−0.07267 (18)	0.27889 (10)	0.0369 (5)	
H10A	0.0969	−0.0780	0.2909	0.044*	
H10B	0.0186	−0.0358	0.3171	0.044*	
C11	0.06233 (10)	−0.29074 (15)	−0.02366 (10)	0.0269 (4)	
H11A	0.0364	−0.3019	0.0196	0.032*	
H11B	0.0277	−0.3109	−0.0602	0.032*	
C12	0.12616 (10)	−0.37655 (16)	−0.02629 (11)	0.0282 (4)	
H12A	0.1533	−0.3665	−0.0690	0.034*	
H12B	0.1601	−0.3611	0.0115	0.034*	
C13	0.09764 (11)	−0.49987 (16)	−0.02144 (11)	0.0293 (4)	
H13A	0.0667	−0.5070	0.0193	0.035*	
H13B	0.0665	−0.5162	−0.0611	0.035*	
C14	0.12156 (11)	−0.12509 (18)	−0.11788 (9)	0.0310 (4)	0.50
H14A	0.0997	−0.1892	−0.1443	0.037*	0.50
H14B	0.1025	−0.0519	−0.1372	0.037*	0.50
C15	0.2010 (2)	−0.1272 (5)	−0.1306 (2)	0.0463 (10)	0.597 (5)
H15A	0.2242	−0.0626	−0.1057	0.056*	0.597 (5)
H15B	0.2214	−0.2007	−0.1129	0.056*	0.597 (5)
C16	0.2200 (4)	−0.1169 (9)	−0.2031 (4)	0.067 (3)	0.597 (5)
H16A	0.1934	−0.0508	−0.2234	0.081*	0.597 (5)
H16B	0.2731	−0.1026	−0.2081	0.081*	0.597 (5)
O4	0.2003 (3)	−0.2228 (4)	−0.2368 (2)	0.0724 (14)	0.597 (5)
H4O	0.165 (3)	−0.196 (3)	−0.271 (2)	0.109*	0.597 (5)
C14A	0.12156 (11)	−0.12509 (18)	−0.11788 (9)	0.0310 (4)	0.50
H14C	0.1303	−0.2044	−0.1353	0.037*	0.50
H14D	0.0838	−0.0887	−0.1467	0.037*	0.50
C15A	0.1934 (3)	−0.0543 (8)	−0.1247 (3)	0.0463 (10)	0.403 (5)
H15C	0.2321	−0.0913	−0.0972	0.056*	0.403 (5)
H15D	0.1856	0.0252	−0.1072	0.056*	0.403 (5)
C16A	0.2184 (6)	−0.0479 (10)	−0.1982 (6)	0.067 (3)	0.403 (5)
H16C	0.1794	−0.0135	−0.2264	0.081*	0.403 (5)
H16D	0.2624	0.0019	−0.2019	0.081*	0.403 (5)
O4A	0.2344 (6)	−0.1593 (11)	−0.2206 (4)	0.090 (4)	0.403 (5)
H4OA	0.229 (7)	−0.155 (4)	−0.2695 (12)	0.135*	0.403 (5)
H1O	0.1497 (16)	0.4974 (19)	0.1345 (15)	0.065 (9)*	
H2O	0.0490 (18)	−0.230 (3)	0.2395 (16)	0.103 (13)*	
H3O	0.1677 (14)	−0.588 (2)	0.0266 (9)	0.047 (7)*	
H2A	0.130 (2)	0.217 (4)	−0.171 (2)	0.098 (13)*	
H2B	0.066 (2)	0.298 (3)	−0.1631 (18)	0.086 (12)*	
H2C	0.1313 (18)	0.309 (3)	−0.1215 (16)	0.068 (9)*	

### Atomic displacement parameters (Å^2^)

**Table:**

	*U*^11^	*U*^22^	*U*^33^	*U*^12^	*U*^13^	*U*^23^
Fe1	0.01608 (17)	0.01771 (17)	0.01507 (17)	0.00023 (12)	0.00013 (12)	0.00109 (12)
Cl1	0.1218 (7)	0.0311 (3)	0.0492 (4)	0.0262 (3)	0.0298 (4)	0.0079 (3)
P1	0.0194 (2)	0.0194 (2)	0.0178 (2)	0.00012 (15)	−0.00260 (16)	0.00053 (16)
P2	0.0183 (2)	0.0216 (2)	0.0196 (2)	0.00237 (16)	−0.00001 (16)	−0.00075 (17)
O1	0.0469 (9)	0.0247 (7)	0.0400 (8)	0.0011 (6)	0.0088 (7)	0.0019 (6)
O2	0.0623 (11)	0.0361 (8)	0.0467 (10)	0.0004 (8)	0.0149 (8)	0.0059 (7)
O3	0.0328 (7)	0.0279 (7)	0.0393 (8)	0.0114 (6)	−0.0014 (6)	−0.0036 (6)
N1	0.0204 (7)	0.0217 (7)	0.0188 (7)	0.0009 (6)	−0.0002 (6)	−0.0011 (6)
C1	0.0281 (9)	0.0263 (9)	0.0212 (8)	−0.0027 (7)	0.0001 (7)	−0.0006 (7)
C2	0.0589 (15)	0.0407 (13)	0.0285 (11)	−0.0206 (12)	0.0064 (11)	0.0062 (10)
C3	0.0185 (8)	0.0289 (9)	0.0284 (9)	−0.0001 (7)	−0.0023 (7)	−0.0027 (7)
C4	0.0197 (8)	0.0294 (9)	0.0242 (9)	0.0051 (7)	−0.0026 (7)	−0.0024 (7)
C5	0.0248 (8)	0.0202 (8)	0.0237 (9)	−0.0014 (7)	−0.0060 (7)	0.0000 (7)
C6	0.0351 (10)	0.0230 (9)	0.0450 (12)	−0.0017 (8)	−0.0191 (9)	0.0013 (9)
C7	0.0402 (11)	0.0278 (10)	0.0374 (11)	−0.0054 (8)	−0.0154 (9)	0.0006 (8)
C8	0.0321 (9)	0.0226 (8)	0.0201 (8)	0.0008 (7)	−0.0056 (7)	0.0034 (7)
C9	0.0529 (13)	0.0278 (10)	0.0219 (9)	0.0086 (9)	0.0019 (9)	0.0006 (8)
C10	0.0547 (13)	0.0342 (11)	0.0218 (9)	0.0022 (9)	−0.0004 (9)	0.0030 (8)
C11	0.0226 (8)	0.0215 (8)	0.0365 (10)	0.0027 (7)	−0.0015 (7)	−0.0006 (8)
C12	0.0226 (9)	0.0253 (9)	0.0367 (10)	0.0047 (7)	0.0002 (8)	−0.0026 (8)
C13	0.0260 (9)	0.0242 (9)	0.0378 (11)	0.0064 (7)	−0.0033 (8)	−0.0034 (8)
C14	0.0340 (10)	0.0380 (11)	0.0212 (9)	0.0044 (8)	0.0048 (8)	−0.0031 (8)
C15	0.0307 (15)	0.077 (3)	0.0314 (15)	0.009 (2)	0.0063 (12)	0.012 (2)
C16	0.045 (2)	0.107 (7)	0.050 (3)	0.016 (5)	0.0194 (18)	0.032 (6)
O4	0.090 (4)	0.082 (3)	0.045 (2)	0.043 (3)	0.024 (2)	0.000 (2)
C14A	0.0340 (10)	0.0380 (11)	0.0212 (9)	0.0044 (8)	0.0048 (8)	−0.0031 (8)
C15A	0.0307 (15)	0.077 (3)	0.0314 (15)	0.009 (2)	0.0063 (12)	0.012 (2)
C16A	0.045 (2)	0.107 (7)	0.050 (3)	0.016 (5)	0.0194 (18)	0.032 (6)
O4A	0.083 (7)	0.143 (10)	0.043 (4)	0.043 (5)	0.005 (4)	−0.008 (5)

### Geometric parameters (Å, °)

**Table:**

Fe1—N1^i^	1.9077 (14)	C8—C9	1.517 (3)
Fe1—N1	1.9077 (14)	C8—H8A	0.9900
Fe1—P1	2.2884 (4)	C8—H8B	0.9900
Fe1—P1^i^	2.2884 (4)	C9—C10	1.529 (3)
Fe1—P2^i^	2.3049 (4)	C9—H9A	0.9900
Fe1—P2	2.3049 (4)	C9—H9B	0.9900
P1—C5	1.8300 (18)	C10—H10A	0.9900
P1—C3	1.8358 (18)	C10—H10B	0.9900
P1—C8	1.8387 (18)	C11—C12	1.531 (2)
P2—C11	1.8341 (19)	C11—H11A	0.9900
P2—C14	1.8388 (19)	C11—H11B	0.9900
P2—C4	1.8409 (18)	C12—C13	1.517 (3)
O1—C7	1.421 (2)	C12—H12A	0.9900
O1—H1O	0.928 (17)	C12—H12B	0.9900
O2—C10	1.415 (3)	C13—H13A	0.9900
O2—H2O	0.976 (18)	C13—H13B	0.9900
O3—C13	1.426 (2)	C14—C15	1.477 (4)
O3—H3O	0.924 (17)	C14—H14A	0.9900
N1—C1	1.140 (2)	C14—H14B	0.9900
C1—C2	1.457 (3)	C15—C16	1.483 (8)
C2—H2A	0.87 (4)	C15—H15A	0.9900
C2—H2B	0.94 (4)	C15—H15B	0.9900
C2—H2C	0.90 (3)	C16—O4	1.438 (10)
C3—C4	1.528 (3)	C16—H16A	0.9900
C3—H3A	0.9900	C16—H16B	0.9900
C3—H3B	0.9900	C16—H4OA	1.40 (2)
C4—H4A	0.9900	O4—H4O	0.98 (2)
C4—H4B	0.9900	O4—H4OA	1.15 (9)
C5—C6	1.531 (2)	C15A—C16A	1.531 (13)
C5—H5A	0.9900	C15A—H15C	0.9900
C5—H5B	0.9900	C15A—H15D	0.9900
C6—C7	1.518 (3)	C16A—O4A	1.390 (15)
C6—H6A	0.9900	C16A—H16C	0.9900
C6—H6B	0.9900	C16A—H16D	0.9900
C7—H7A	0.9900	O4A—H4O	1.67 (3)
C7—H7B	0.9900	O4A—H4OA	0.98 (2)
			
N1^i^—Fe1—N1	180.0	C9—C8—H8B	107.7
N1^i^—Fe1—P1	91.51 (4)	P1—C8—H8B	107.7
N1—Fe1—P1	88.49 (4)	H8A—C8—H8B	107.1
N1^i^—Fe1—P1^i^	88.49 (4)	C8—C9—C10	112.18 (17)
N1—Fe1—P1^i^	91.51 (4)	C8—C9—H9A	109.2
P1—Fe1—P1^i^	180.00 (3)	C10—C9—H9A	109.2
N1^i^—Fe1—P2^i^	89.90 (4)	C8—C9—H9B	109.2
N1—Fe1—P2^i^	90.10 (4)	C10—C9—H9B	109.2
P1—Fe1—P2^i^	95.813 (16)	H9A—C9—H9B	107.9
P1^i^—Fe1—P2^i^	84.187 (16)	O2—C10—C9	112.52 (17)
N1^i^—Fe1—P2	90.10 (4)	O2—C10—H10A	109.1
N1—Fe1—P2	89.90 (4)	C9—C10—H10A	109.1
P1—Fe1—P2	84.187 (16)	O2—C10—H10B	109.1
P1^i^—Fe1—P2	95.813 (16)	C9—C10—H10B	109.1
P2^i^—Fe1—P2	180.00 (3)	H10A—C10—H10B	107.8
C5—P1—C3	104.19 (8)	C12—C11—P2	116.53 (13)
C5—P1—C8	104.85 (8)	C12—C11—H11A	108.2
C3—P1—C8	99.48 (9)	P2—C11—H11A	108.2
C5—P1—Fe1	120.80 (6)	C12—C11—H11B	108.2
C3—P1—Fe1	105.32 (6)	P2—C11—H11B	108.2
C8—P1—Fe1	119.09 (6)	H11A—C11—H11B	107.3
C11—P2—C14	103.44 (9)	C13—C12—C11	109.88 (15)
C11—P2—C4	102.82 (9)	C13—C12—H12A	109.7
C14—P2—C4	105.10 (9)	C11—C12—H12A	109.7
C11—P2—Fe1	118.75 (6)	C13—C12—H12B	109.7
C14—P2—Fe1	116.66 (7)	C11—C12—H12B	109.7
C4—P2—Fe1	108.46 (6)	H12A—C12—H12B	108.2
C7—O1—H1O	108.1 (19)	O3—C13—C12	112.82 (16)
C10—O2—H2O	110 (2)	O3—C13—H13A	109.0
C13—O3—H3O	105.3 (17)	C12—C13—H13A	109.0
C1—N1—Fe1	178.43 (15)	O3—C13—H13B	109.0
N1—C1—C2	178.4 (2)	C12—C13—H13B	109.0
C1—C2—H2A	110 (3)	H13A—C13—H13B	107.8
C1—C2—H2B	108 (2)	C15—C14—P2	120.8 (2)
H2A—C2—H2B	113 (3)	C15—C14—H14A	107.1
C1—C2—H2C	112 (2)	P2—C14—H14A	107.1
H2A—C2—H2C	111 (3)	C15—C14—H14B	107.1
H2B—C2—H2C	104 (3)	P2—C14—H14B	107.1
C4—C3—P1	106.90 (12)	H14A—C14—H14B	106.8
C4—C3—H3A	110.3	C16—C15—C14	113.3 (4)
P1—C3—H3A	110.3	C16—C15—H15A	108.9
C4—C3—H3B	110.3	C14—C15—H15A	108.9
P1—C3—H3B	110.3	C16—C15—H15B	108.9
H3A—C3—H3B	108.6	C14—C15—H15B	108.9
C3—C4—P2	108.88 (12)	H15A—C15—H15B	107.7
C3—C4—H4A	109.9	O4—C16—C15	108.9 (6)
P2—C4—H4A	109.9	O4—C16—H16A	109.9
C3—C4—H4B	109.9	C15—C16—H16A	109.9
P2—C4—H4B	109.9	O4—C16—H16B	109.9
H4A—C4—H4B	108.3	C15—C16—H16B	109.9
C6—C5—P1	115.37 (12)	H16A—C16—H16B	108.3
C6—C5—H5A	108.4	O4—C16—H4OA	48 (4)
P1—C5—H5A	108.4	C15—C16—H4OA	157 (3)
C6—C5—H5B	108.4	H16A—C16—H4OA	85.2
P1—C5—H5B	108.4	H16B—C16—H4OA	80.6
H5A—C5—H5B	107.5	C16—O4—H4O	102.7 (19)
C7—C6—C5	112.10 (16)	C16—O4—H4OA	64.3 (16)
C7—C6—H6A	109.2	H4O—O4—H4OA	73 (5)
C5—C6—H6A	109.2	C16A—C15A—H15C	109.4
C7—C6—H6B	109.2	C16A—C15A—H15D	109.4
C5—C6—H6B	109.2	H15C—C15A—H15D	108.0
H6A—C6—H6B	107.9	O4A—C16A—C15A	108.8 (8)
O1—C7—C6	109.56 (17)	O4A—C16A—H16C	109.9
O1—C7—H7A	109.8	C15A—C16A—H16C	109.9
C6—C7—H7A	109.8	O4A—C16A—H16D	109.9
O1—C7—H7B	109.8	C15A—C16A—H16D	109.9
C6—C7—H7B	109.8	H16C—C16A—H16D	108.3
H7A—C7—H7B	108.2	C16A—O4A—H4O	105.5 (16)
C9—C8—P1	118.28 (13)	C16A—O4A—H4OA	104 (2)
C9—C8—H8A	107.7	H4O—O4A—H4OA	49 (8)
P1—C8—H8A	107.7		
			
N1^i^—Fe1—P1—C5	−126.74 (8)	Fe1—P1—C3—C4	−52.35 (13)
N1—Fe1—P1—C5	53.26 (8)	P1—C3—C4—P2	52.78 (15)
P2^i^—Fe1—P1—C5	−36.69 (7)	C11—P2—C4—C3	−157.24 (13)
P2—Fe1—P1—C5	143.31 (7)	C14—P2—C4—C3	94.80 (14)
N1^i^—Fe1—P1—C3	115.91 (8)	Fe1—P2—C4—C3	−30.65 (14)
N1—Fe1—P1—C3	−64.09 (8)	C3—P1—C5—C6	−45.93 (17)
P2^i^—Fe1—P1—C3	−154.03 (6)	C8—P1—C5—C6	58.13 (16)
P2—Fe1—P1—C3	25.97 (6)	Fe1—P1—C5—C6	−163.84 (12)
N1^i^—Fe1—P1—C8	5.56 (8)	P1—C5—C6—C7	−170.84 (15)
N1—Fe1—P1—C8	−174.44 (8)	C5—C6—C7—O1	−64.9 (2)
P2^i^—Fe1—P1—C8	95.61 (7)	C5—P1—C8—C9	53.06 (17)
P2—Fe1—P1—C8	−84.39 (7)	C3—P1—C8—C9	160.61 (15)
N1^i^—Fe1—P2—C11	24.40 (9)	Fe1—P1—C8—C9	−85.84 (16)
N1—Fe1—P2—C11	−155.60 (9)	P1—C8—C9—C10	−179.89 (14)
P1—Fe1—P2—C11	115.91 (8)	C8—C9—C10—O2	−70.4 (2)
P1^i^—Fe1—P2—C11	−64.09 (8)	C14—P2—C11—C12	65.29 (17)
N1^i^—Fe1—P2—C14	149.30 (9)	C4—P2—C11—C12	−43.92 (17)
N1—Fe1—P2—C14	−30.70 (9)	Fe1—P2—C11—C12	−163.61 (12)
P1—Fe1—P2—C14	−119.19 (8)	P2—C11—C12—C13	−178.05 (14)
P1^i^—Fe1—P2—C14	60.81 (8)	C11—C12—C13—O3	−175.41 (17)
N1^i^—Fe1—P2—C4	−92.34 (8)	C11—P2—C14—C15	−97.4 (3)
N1—Fe1—P2—C4	87.66 (8)	C4—P2—C14—C15	10.1 (3)
P1—Fe1—P2—C4	−0.84 (7)	Fe1—P2—C14—C15	130.3 (3)
P1^i^—Fe1—P2—C4	179.16 (7)	P2—C14—C15—C16	−179.9 (5)
C5—P1—C3—C4	179.56 (12)	C14—C15—C16—O4	−71.1 (7)
C8—P1—C3—C4	71.49 (14)		

Symmetry codes: (i) −*x*, −*y*, −*z*.

### Hydrogen-bond geometry (Å, °)

**Table:**

*D*—H···*A*	*D*—H	H···*A*	*D*···*A*	*D*—H···*A*
O1—H1O···Cl1	0.93 (2)	2.13 (2)	3.0493 (16)	173 (3)
O2—H2O···Cl1^ii^	0.98 (2)	2.23 (2)	3.1777 (19)	164 (3)
O3—H3O···O1^ii^	0.92 (2)	1.84 (2)	2.741 (2)	164 (2)
O4—H4O···Cl1^iii^	0.98 (2)	1.95 (2)	2.931 (6)	177 (3)
O4A—H4OA···Cl1^iii^	0.98 (2)	2.84 (11)	3.490 (10)	125 (9)

Symmetry codes: (ii) *x*, *y*−1, *z*; (iii) *x*, −*y*+1/2, *z*−1/2.
